# TAK228 enhances antitumor activity of eribulin in triple negative breast cancer

**DOI:** 10.18632/oncotarget.27082

**Published:** 2019-08-20

**Authors:** Nicci Owusu-Brackett, Kurt W. Evans, Argun Akcakanat, Erkan Yuca, Coya Tapia, Yasmeen Qamar Rizvi, Ecaterina Ileana Dumbrava, Filip Janku, Funda Meric-Bernstam

**Affiliations:** ^1^ Department of Surgical Oncology, The University of Texas MD Anderson Cancer Center, Houston, TX 77030, USA; ^2^ Current address: Department of General Surgery, The University of Texas Health San Antonio, San Antonio, TX 78229, USA; ^3^ Department of Investigational Cancer Therapeutics, The University of Texas MD Anderson Cancer Center, Houston, TX 77030, USA; ^4^ Department of Translational Molecular Pathology, The University of Texas MD Anderson Cancer Center, Houston, TX 77030, USA; ^5^ Department of Breast Surgical Oncology, The University of Texas MD Anderson Cancer Center, Houston, TX 77030, USA; ^6^ The Sheikh Khalifa Bin Zayed Al Nahyan Institute for Personalized Cancer Therapy, The University of Texas MD Anderson Cancer Center, Houston, TX 77030, USA

**Keywords:** breast cancer, TAK228, PI3K, PTEN, TNBC

## Abstract

**Background:** Phosphatase and tensin homologue deleted from chromosome 10 (PTEN) negatively regulates the phosphatidylinositol 3-kinase (PI3K)/AKT/mTOR pathway. Triple negative breast cancers (TNBC) are often PTEN-deficient, making mTOR a compelling target. We evaluated the efficacy of catalytic mTOR inhibitor TAK228 alone and in combination with eribulin in TNBC.

**Results:** Five of eight triple negative breast cell lines were sensitive to TAK228, independent of PIK3CA/PTEN status. Western blotting demonstrated inhibition of mTORC1/2 signaling as demonstrated by decreased phospho-AKT, phospho-S6 and phospho-4EBP1. *In vitro*, TAK228 was synergistic with eribulin in all eight TNBC cell lines. The combination of TAK228 and eribulin did not enhance apoptosis but increased G2/M growth arrest. *In vivo*, TAK228 led to modest growth inhibition in TNBC patient-derived xenografts (PDXs) with no tumor regression observed. In two TNBC PDXs with PTEN loss, one with intrinsic eribulin sensitivity, another eribulin resistance, TAK228 in combination with eribulin did not enhance *in vivo* efficacy. In a third PTEN-negative TNBC model, eribulin alone achieved disease stabilization, but the combination of TAK228 and eribulin led to significantly smaller tumor volumes compared to eribulin alone (*p* < 0.001).

**Methods:** We tested *in vitro* efficacy of TAK228 in a panel of TNBC cell lines with cell proliferation assays. *In vivo* antitumor efficacy of TAK228 was evaluated alone and in combination with eribulin.

**Conclusion:** TAK228 enhances the antitumor efficacy of eribulin in TNBC models *in vitro*, and enhanced *in vivo* activity in selected models. Further study is needed to determine the potential of this combination, and optimal patient selection strategies.

## INTRODUCTION

Phosphatidylinositol 3-kinase (PI3K)-AKT-mTOR pathway regulates cell metabolism, proliferation and migration [1]. Deregulation of this signaling pathway is common in triple negative breast cancers (TNBC). Mechanisms such as loss of phosphatase tensin homolog deleted from chromosome 10 (PTEN) or mutational activation of *PIK3CA* have been reported to increase activation of this pathway [2, 3]. PTEN negatively regulates the PI3K-AKT-mTOR pathway, which maintains balanced cell differentiation, proliferation and survival. Mutations or deletions in *PTEN*, loss of copy number, epigenetic regulation and downregulation of PTEN protein by miRNA can result in loss of PTEN protein. Loss of PTEN results in amplified cell proliferation and tumor initiation across a variety of solid tumors, including breast cancer [4, 5]. Furthermore, loss of PTEN in TNBC has been associated with a poor outcome [6]. PI3K activation leads to phosphorylation and activation of AKT, which in turn can activate downstream mTOR. Thus, targeting the PI3K-AKT-mTOR pathway in TNBC with PTEN loss with mTOR inhibitors is compelling.

mTOR serine/threonine kinase forms 2 distinct complexes: mTORC1 and mTORC2. mTORC1 regulates translation via phosphorylation of ribosomal protein S6 kinase-1 (p70S6K1) and 4E-binding protein 1 (4E-BP1) while mTORC2 promotes cell survival via phosphorylation of AKT at Ser473 [7]. TAK228 is a dual inhibitor of mTORC1 and mTORC2. In previous work we have shown that TAK228 has antitumor efficacy in cell lines sensitive to allosteric mTOR inhibitor rapamycin, as well as cell lines with intrinsic and acquired resistance to rapamycin [8]. Garcia et al showed that dual mTORC1 and mTORC2 inhibition in combination with anti-HER2 therapy resulted in suppression of cancer cell growth in HER2-positive breast cancer; however, the efficacy of TAK228 in TNBC with and without PTEN-loss, in combination therapy has not been evaluated [9]. Therefore, we sought to determine the antitumor efficacy of TAK228 in TNBC cell lines with varying backgrounds as a single agent and in combination with standard chemotherapeutic agents. *In vivo* confirmation of *in vitro* identified combinations was performed.

## RESULTS

### TAK228 inhibits Akt/mTOR signaling

**Figure 1:**
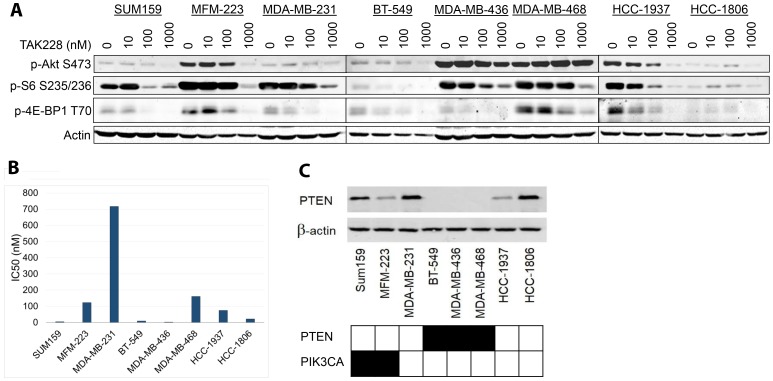
Effects of TAK228 on cell proliferation *in vitro.* (**A**) Eight triple negative breast cancer cell lines were treated for 48 hours with DMSO or varying doses of TAK228 ranging from 10 nM to 1000 nM, a physiologically achievable dose. Cells were lysed and blotted with the indicated antibodies. (**B**) The same panel of cell lines in (A) were treated with TAK228 at 6 concentrations based on a five-fold dilution series (range 0 to 10 μM). Cell growth was measured after 72 hours of treatment using the sulforhodamine B assay, and half-maximal inhibitory concentration (IC_50_) was then calculated using isobologram curves. Sensitivity was defined as IC_50_ < 80 nM. (**C**) The same panel of cell lines (A) were lysed and blotted with indicated antibodies. PTEN and PIK3CA mutation statuses demonstrated with black indicating mutation and white indicating wildtype.

To evaluate the mechanism of action of TAK228, we assessed its effect on the Akt/mTOR signaling in eight breast cancer cell lines (Figure 1A). We treated the TNBC cell lines with varying doses of TAK228, ranging from 10 nM to 1000 nM, or DMSO for 48 hours. S6 and 4E-BP1 phosphorylation was inhibited in all cell lines but HCC-1806, which had very low expression of these markers and the result was not clear. Akt phosphorylation was decreased in six cell lines, whereas a dose related increase was observed in MDA-MB-468 cell line.

### TAK228 has antitumor efficacy as a single agent in TNBC cells *in vitro*

To evaluate the antitumor efficacy of TAK228 in TNBC cell lines, we tested TAK228 sensitivity in a panel of eight TNBC lines with varying PTEN/PIK3CA genotypes. Maximum plasma concentration obtainable in patients receiving TAK228 with 4 mg daily was 36.425 ng/mL for fasted state, which is equivalent to 116 nM [10]. Given the clinically achievable dose, sensitivity to TAK228 was defined as IC_50_ less than 80 nM. Cell lines were treated with DMSO or TAK228 for 72 hours. Cell growth was measured using SRB colorimetric assay. Sensitivity was evaluated by calculating the IC_50_ using isobologram curves. Five of eight triple negative breast cancer cell lines were sensitive to TAK228 (Figure 1B). To assess the effects of PI3K/PTEN expression on sensitivity to TAK228, the Catalogue of Somatic Mutations in Cancer (COSMIC) database was used to determine the mutation status of each of the eight TNBC cell lines. Western blots were performed on all eight cell lines to determine baseline PTEN protein expression level (Figure 1C). The TNBC cell line we analyzed seemed to be enriched for PI3K/PTEN pathway alterations, but sensitivity to TAK228 did not seem to be associated with PI3K or PTEN status.

### TAK228 in combination with eribulin has enhanced antitumor efficacy *in vitro*

**Figure 2:**
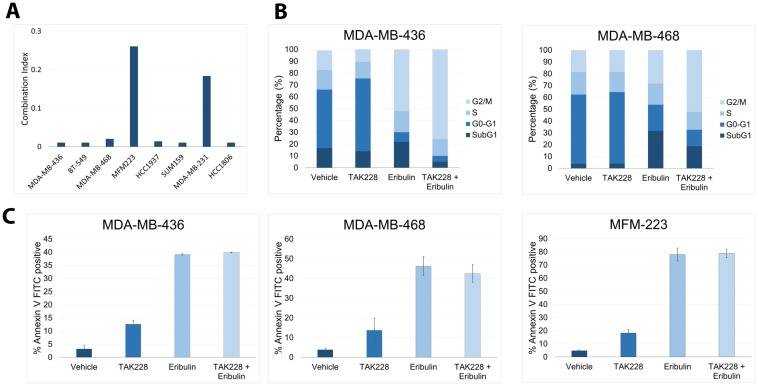
Effects of TAK228 in combination with eribulin *in vitro*. Eight triple-negative breast cancer cell lines were treated with TAK228 in combination with eribulin. Cell growth was measured after 72 hours of treatment using the sulforhodamine B assay, and the combination index (CI) was then calculated using the method of Chou and Talalay. A CI value <0.8 indicates synergism, a value equal to 1 indicates additive effect, and a CI significantly>1.2 indicates antagonism. (**B**) MDA-MB-436 and MDA-MB-468 cells were treated with the vehicle, 50 nM TAK228, 10 nM eribulin or TAK228 in combination with eribulin. After 48 hours, cell cycle was determined with propidium iodide by fluorescence-activated cell sorting (FACS) analysis. (**C**) MDA-MB-436, MDA-MB-468 and MFM-223 cells were treated with the vehicle, 50 nM TAK228, 10 nM eribulin or TAK228 in combination with eribulin. After 48 hours, annexin-V-positive cells were determined by FACS analysis.

To determine the effects of TAK228 in combination with eribulin, eight TNBC cell lines were treated with serial concentration dilutions of TAK228 in combination with serial concentration dilutions of the eribulin. After 72 hours of treatment, growth inhibition was assessed with SRB assay, and IC_50_ was calculated for single agent treatment alone and the combination. Combination index (CI) values were then calculated using the method of Chou and Talalay, where a CI value <0.8 indicates synergism, 0.8 to 1.2 indicates additive effect and a CI greater than 1.2 indicates antagonism [11, 12]. Synergy was observed in all eight cell lines (Figure 2A).

### TAK228 in combination with eribulin increases G2/M growth arrest

The effect of TAK228 on cell-cycle progression was evaluated in breast cancer cell lines. The cells were treated with the vehicle, 50 nM TAK228 50 nM, 10 nM eribulin or TAK228 in combination with eribulin for 48 hours and subsequently harvested. Percentage of cells in G1, S and G2-M phases of the cell cycle were determined by flow cytometry using propidium iodide. In both cell lines TAK228 in combination with eribulin resulted in an increase in G2/M growth arrest (Figure 2B). Next, apoptosis analysis was performed and the percentage of annexin V-positive cells were determined with fluorescence-activated cell sorting (FACS). TAK228 in combination with eribulin does not enhance apoptosis (Figure 2C).

### TAK228 has modest single agent antitumor efficacy in patient-derived xenografts

**Figure 3:**
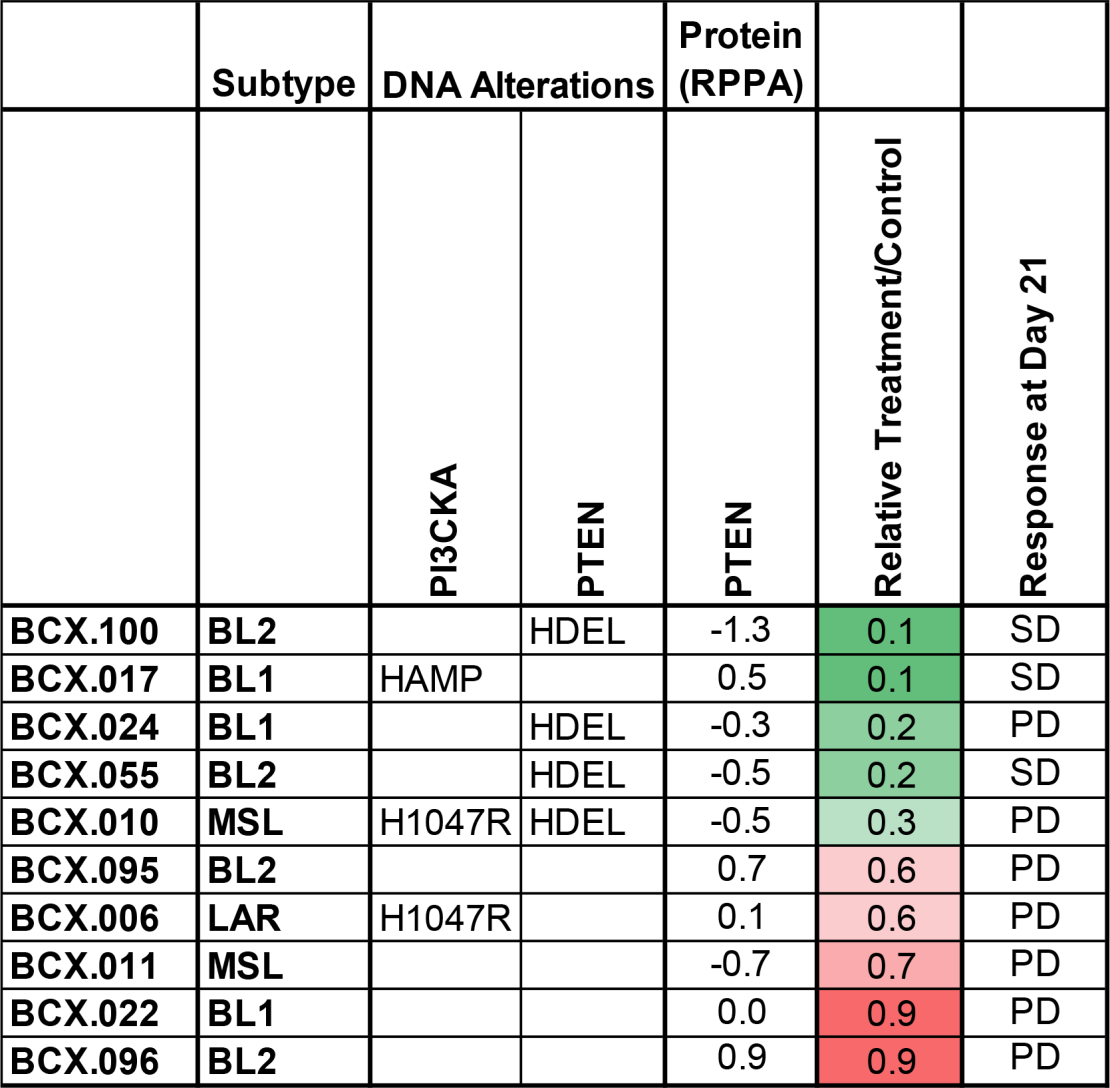
Effects of TAK228 in patient derived xenografts. Ten patient derived xenografts were treated with vehicle or TAK228 1 mg/kg daily. Genomic alterations, PTEN protein expression and molecular subtypes of TNBCs are presented. Relative growth calculated as median change in treatment tumor volume/median change in control tumor volume at the first measurement at which median of control tumors was twice the median starting volume (green reflects greater growth inhibition). (BL1 = basal-like 1, BL2 = basal-like 2, MSL = mesenchymal stem-like, LAR = luminal androgen receptor; HAMP ≥ 4 gene copies; HDEL ≤ 1 gene copies; RPPA = Reverse Phase Protein Array; PD = progressive disease, SD = stable disease).

We evaluated the single agent efficacy of TAK228 in eight TNBC patient-derived xenografts of varying PTEN and PIKCA genotypes. TAK228 led to modest growth inhibition of five TNBC PDX models with relative treatment-to-control ratio of less than or equal to 0.5. Inhibition was noted to be independent of PI3K or PTEN status; however, progression of disease was noted in all but one model at day 21 (Figure 3, Supplementary Figure 1). Notably all four models with *PTEN* deletion and two of three models with PIK3CA alterations had a treatment-to-control ratio of less than or equal to 0.5, suggesting that TAK228 had growth inhibitory effect. However, ultimately all but one model progressed by day 21, while the BCX.055 model (with PTEN loss) had stable disease.

### TAK228 in combination with eribulin has enhanced antitumor efficacy *in vivo*

**Figure 4:**
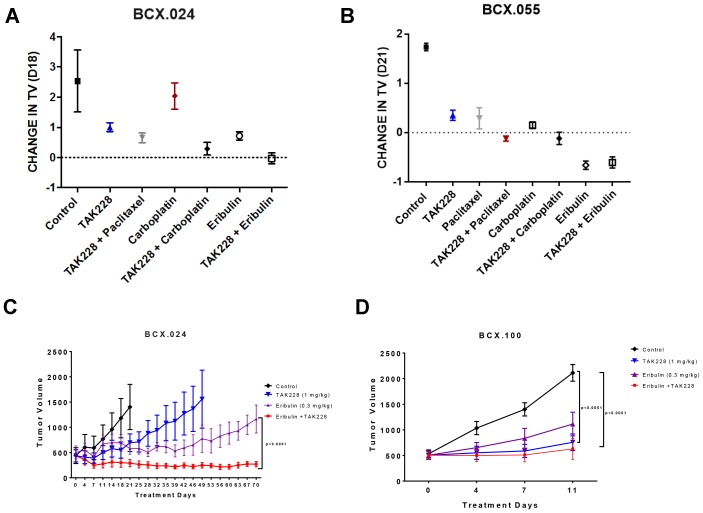
Effects of TAK228 in combination with chemotherapy *in vivo*. PTEN loss PDX BCX.024 (**A**) and BCX.055 (**B**) were treated with TAK228 and a variety of chemotherapeutic agents to screen for potential combination therapies (*n =* 2-3). (**C**) BCX.024 and (**D**) BCX.100 xenografts were treated with vehicle (*n =* 5; *n =* 4), TAK228 1 mg/kg daily (*n =* 5; *n =* 4), eribulin 0.3 mg/kg weekly (*n =* 5; *n =* 5) or TAK228 in combination with eribulin (*n =* 4; *n =* 4). Values are presented as mean ± SEM of tumor volume. P-value shown are multiple comparisons test on final day of possible comparison.

We hypothesized that TAK228 may enhance *in vivo* antitumor efficacy of standard chemotherapeutic agents. This was evaluated in a signal-seeking experiment, in two PTEN-deficient PDXs, treated with either vehicle, TAK228, paclitaxel, eribulin, carboplatin or TAK228 in combination with each of the chemotherapeutic agents, with *n =* 2–3 for each group. Treatments were started once tumors reached at least 100 mm^3^. In the BCX.024 model, neither eribulin nor TAK228 alone achieved stable disease, but TAK228 in combination with eribulin resulted in growth stabilization (tumor volume of -3%; Figure 4A, Supplementary Figure 2). In the eribulin-sensitive model BCX.055, eribulin alone achieved tumor regression with a change in tumor volume of (−60%) but TAK228 did not enhance efficacy of eribulin (Figure 4B, Supplementalry Figure 2).

To confirm the antitumor efficacy of TAK228 with eribulin, we performed a larger PDX cohort study (*n =* 4−5) in the BCX.024 model as well as in another PTEN loss model BCX.100. PDXs were treated with vehicle, TAK228 1 mg/kg daily, eribulin 0.3 mg/kg weekly or TAK228 in combination with eribulin. Neither group achieved tumor stabilization. In BCX.024, TAK228 in combination with eribulin led to tumor regression that was maintained for the 70 days PDXs were treated (-38% at day 70), with significantly greater growth inhibition compared with eribulin alone (*p *< 0.0001; Figure 4C). In BCX.100, TAK228 alone as well as in combination with eribulin led to statistically significant growth inhibition compared to vehicle (*p * <0.01 for both treatment groups), but eribulin did not enhance TAK228’s efficacy (Figure 4D).

### Proliferation, apoptosis and mTOR pathway inhibition in BCX.024 patient-derived xenograft model

Immunohistochemical analysis revealed a lower proliferation marker Ki-67 in xenografts treated with TAK228 as a single agent and in combination with eribulin (mean percentages of positive cells: control 65%, TAK228 29.8%, eribulin 76%, and TAK228 and eribulin combination 33.2%) (Supplementary Figure 3). There was no significant change in apoptosis marker cleaved caspase 3, and mTOR pathway markers p-S6 (S235/236) and p-S6 (S240/244) (Supplementary Figure 3).

## DISCUSSION

TNBC constitutes approximately 15−20% of breast cancer patients and is associated with a poor prognosis [13]. TNBC patients with residual disease following neoadjuvant chemotherapy are at high risk of relapse and have few options upon recurrence [14]. Therefore, there is a great need for superior therapeutic options for TNBC. PTEN loss has been commonly reported in TNBC. TAK228 is a dual inhibitor of the mTORC1 and mTORC2, both of which have increased activity in PTEN-deficient tumors. The inhibitory effect of TAK228 is specific to the mTOR pathway. Silencing or re-activating mTOR target 4E-BP1 regulates the ability of TAK228 to decrease expression of downstream invasion mRNAs and in causing apoptosis [15, 16]. We therefore tested the efficacy of TAK228 alone and in combination with chemotherapy in TNBC models with and without PTEN loss.

Five of eight TNBC cell lines were sensitive to TAK228 and this appeared to be independent of PIK3CA/PTEN status. Our panel was enriched for PI3K pathway alterations and we expected a higher TAK228 efficacy compared to models without any pathway aberrations. It was likely that co-alterations such as the KRAS mutation found in MDA-MB-231 limited the activity. We did not do an in-depth analysis but the hotspot mutations in HCC-1806 cell line could not explain why it was very sensitive to TAK228 [17]. Interestingly, Akt phosphorylation was increased in a dose-dependent manner in MDA-MB-468 (Figure 1A). We had observed a similar activation in our previous work [8]. The weak signal did not allow us to make a definitive statement in MDA-MB-231, where p-Akt levels seem to be stable under treatment. These two were the most resistant cell lines and further work is needed to assess the *in vitro* and *in vivo* mediators of TAK228 sensitivity.

Although some single agent growth-inhibitory activity was noted in TNBC PDX models, most models progressed, demonstrating the need for combination therapy, even in models with PI3K/PTEN alterations. However, when we tested the efficacy of TAK228 with common chemotherapies used in breast cancer therapy, we noted that the combination of eribulin and TAK228 enhanced antitumor efficacy. *In vitro* evaluation demonstrated synergy of TAK228 in combination with eribulin in all TNBC cell lines evaluated. Our findings are of interest as there is emerging data demonstrating the role of targeting PI3K/Akt signaling in TNBC. The LOTUS trial investigated the addition of AKT inhibitor ipatasertib to paclitaxel as first-line therapy for metastatic/advanced TNBC [18]. Median progression-free survival in the intention-to-treat population was 6.2 months with ipatasertib versus 4.9 months with placebo (stratified hazard ratio [HR] 0.60, 95% CI 0.37-0.98; *p =* 0.037). Pre-specified analyses in the 42 patients with *PIK3CA/AKT1/PTEN*-altered tumors showed a median progression-free survival of 9.0 months with ipatasertib versus 4.9 months with placebo (non-stratified HR 0.44, 95% CI 0.20–0.99, log-rank *p =* 0.041), while PFS was not significantly different in patients with *PIK3CA/AKT1/PTEN-*non-altered tumors. Additionally, the PAKT trial reported that the addition of AZD5363 to 1st-line paclitaxel therapy for TNBC resulted in significantly longer PFS (5.9 months vs. 4.2 months) and OS (19.1 months vs. 12.6 months) [19]. Thus our data suggest that TAK228, like AKT inhibitors, may have efficacy in combination with chemotherapy for TNBC.

Our study has several limitations. We focused on TNBC, thus we have not assessed efficacy of TAK228 in estrogen receptor (ER)+ or HER2+ tumors. Similarly, we did our *in vitro* screen in TNBC models, and almost all had PI3K pathway alterations. It is possible that if we expanded our work to cell lines without PI3K alterations, there would have been less TAK228 efficacy, and an association with PI3K/PTEN status and TAK228 sensitivity would have emerged. BCX.024 is a model generated from residual tumor after neoadjuvant chemotherapy, and the model is resistant to paclitaxel: it is possible that models sensitive to paclitaxel may have shown enhanced anti-tumor efficacy with the TAK228 paclitaxel combination. Although TAK228 as a single agent or in combination inhibited Ki-67 expression (Supplementary Figure 3), this decrease was not significant. We did not have paraffin blocks of all the xenograft tumors and small sample size had a negative impact on statistical analysis.

Taken together, our results demonstrate that TAK228 is efficacious *in vitro*, and has some single-agent growth inhibitory effect *in vivo*. To have greater clinical impact, further work is needed to identify models where TAK228 would demonstrate greater single agent efficacy, with durable tumor regression. We here demonstrate *in vitro* and *in vivo* that TAK228 in combination with eribulin is a promising novel therapy for TNBC.

## MATERIALS AND METHODS

### Cell lines and culture

Breast cancer cell lines were obtained from the American Tissue Culture Collection: BT-549, HCC-1806, HCC-1937, MDA-MB-231, MDA-MB-436, MDA-MB-468, SUM-159PT and MFM-223. Cells were cultured in Dulbecco’s modified Eagle’s medium/F-12 supplemented with 10% fetal bovine serum at 37°C in a humidified incubator containing 5% CO_2_.

### *In vitro* reagents and drugs

TAK228, paclitaxel and carboplatin were obtained from Selleck Chemicals, and eribulin was acquired from MD Anderson Cancer Center’s pharmacy. Dimethyl sulfoxide (DMSO) was purchased from Sigma-Aldrich. All drugs were dissolved in DMSO.

### Cell growth assay

Cells were seeded in 96-well plates at densities of 5000 to 10000 cells per well depending on growth characteristics of each cell line. After adhering overnight, titrating concentrations of the designated drug were added to the wells in triplicates and incubated at 37°C for 72 hours. Antiproliferative activity was evaluated by sulforhodamine B (SRB) assay. The half maximal inhibitory concentration (IC_50_) and combination index (CI) were determined from dose-response curves generated using GraphPad Prism v6.05 software. All experiments were repeated at least three times.

### Western blot analysis

Cells were washed with cold PBS and lysed in Laemmli buffer. The protein was quantified using Pierce BCA protein assay Kit (ThermoFisher) before loading to the gel. After SDS-PAGE, the protein was transferred to a 0.2 μm nitrocellulose membrane (Bio-Rad Laboratories). Membranes were blocked with 0.1% casein in TBS. Immunoblotting was performed with the following antibodies: pAkt S473, pS6 S235/236, p4E-BP1 T70, PTEN, and β-actin. After a washing step, membranes were incubated with secondary antibodies. The immunoblots were visualized using the Odyssey IR imaging system (Li-Cor Biosciences). Representative blots of at least 2 independent experiments are shown.

### Cell cycle and apoptosis assays

Cells were plated and allowed to attach to the petri dish overnight. The following day, cells were treated with DMSO, TAK228, eribulin or combination in triplicates. After 48 hours, floating and attached cells were collected. DNA content was determined in flow cytometry using propidium iodide (Roche) following manufacturer’s protocol. Apoptosis was identified by using the Annexin V apoptosis kit (Roche) according to the manufacturer’s protocol. Samples were analyzed by flow cytometry at The Flow Cytometry and Cellular Imaging (FCCI) Core Facility at MD Anderson.

### Immunohistochemistry

IHC stain was performed on 4 μm thick tissue sections using the following antibodies and dilutions: mouse monoclonal anti-Ki-67, clone MIB-1 (DAKO/Agilent, Santa Clara, CA, USA; cat# M7240) with a dilution of 1:100, rabbit polyclonal anti-cleaved caspase-3 (Cell Signaling, Danvers, MA, USA; cat# 9661) with a dilution of 1:100, rabbit monoclonal anti-pS6 (S235/236), clone D57.2.2E and rabbit polyclonal anti-pS6 (S240/244) (Cell Signaling, Danvers, MA, USA, cat# 4858 and cat# 2215) both pS6 antibodies were used with a dilution of 1:100. All stains were evaluated by a pathologist (CT). For Ki-67 and cleaved caspase-3 the fraction of positive cells was estimated (percentage) and for the pS6 antibodies the percentage of positivity and the staining intensity was estimated resulting in an H-score (0–300).

### *In vivo* studies

All animal experiments were approved by the Institutional Animal Care and Use Committee of MD Anderson. Generation of TNBC PDX models, genomic and reverse phase protein assay data have been previously described [20, 21]. Tumors were implanted into female BALB/c *nu*/*nu* mice, 6 to 8 weeks old, under isoflurane anesthesia. A skin incision (approximately 0.3 cm) was made with a subcutaneous pocket on the mid back. One tumor piece (approximately 27 mm^3^) was inserted into a pocket and the skin was then closed. The mice were treated when the tumor diameter reached at least 200 mm^3^. The mice were killed when the diameter reached 1.5 cm, and the individual relative tumor volume (RTV) was measured. RTV was defined as Vx/V1, where Vx is the volume in mm^3^ at a given time and V1 is the volume at the start of treatment [22, 23].

Paclitaxel, eribulin and carboplatin were purchased from the MD Anderson pharmacy. TAK228 (MLN0128) was purchased from ChemieTek. Doses of paclitaxel (10 mg/kg, i.v. weekly), eribulin (1 mg/kg, i.v. weekly) and carboplatin (75 mg/kg, i.p. weekly) were diluted to appropriate volume in PBS prior to administering to mice. TAK228 (1 mk/kg daily) was dissolved in NVP and diluted in 5% polyvinylpyrrolidone (PVP) in water. Treatment testing was performed using subcutaneous implantation in female athymic nude mice. Tumor volume (TV) was calculated by the formula: TV (mm^3^) = ((width)^2^ × length)/2. Change TV from baseline was calculated as (TV DayX − TVDay0)/TVDay0.

### Statistical analysis

For immunohistochemistry studies, Kruskal Wallis followed by Dunn’s multiple comparisons test was performed to compare multiple groups. For *in vivo* studies, the statistical analyses were performed by comparing RTV in the treatment arms with RTV in the vehicle arm. Tumor growth inhibition ratios (T/C: treatment/control) were calculated using the formula:

[(Median tumor volume of treated group)/(Median tumor volume of control group)] × 100.

Activity was defined as a T/C ratio <40% [24, 25]. Pairwise *t* tests were adjusted by FDR method. The Tukey and FDR methods were used to adjust for multiplicities. Data was presented as means ± SEM.

## SUPPLEMENTARY MATERIALS



## Abbreviations

PI3K: phosphatidylinositol 3-kinase
RPPA: reverse phase proteomic array
PTEN: phosphatase tensin homolog deleted from chromosome 10
CI: combination index
DMSO: dimethyl sulfoxide
FACS: fluorescence-activated cell sorting
IC_50_: half maximal inhibitory concentration
PDX: patient-derived xenograft
SRB: sulforhodamine B
T/C ratio: tumor growth inhibition ratio
TNBC: triple negative breast cancer
