# Supplementary material for: TAK228 enhances antitumor activity of eribulin in triple negative breast cancer
Source: Oncotarget. 2019 Aug 20;10(49):5011–9. doi: 10.18632/oncotarget.27082 (PMC6707945; doi:10.18632/oncotarget.27082)
Supplement: Supplementary file 1 [file oncotarget-10-5011-s001.pdf]

TAK228 enhances antitumor activity of eribulin in triple negative breast cancer

SUPPLEMENTARY MATERIALS

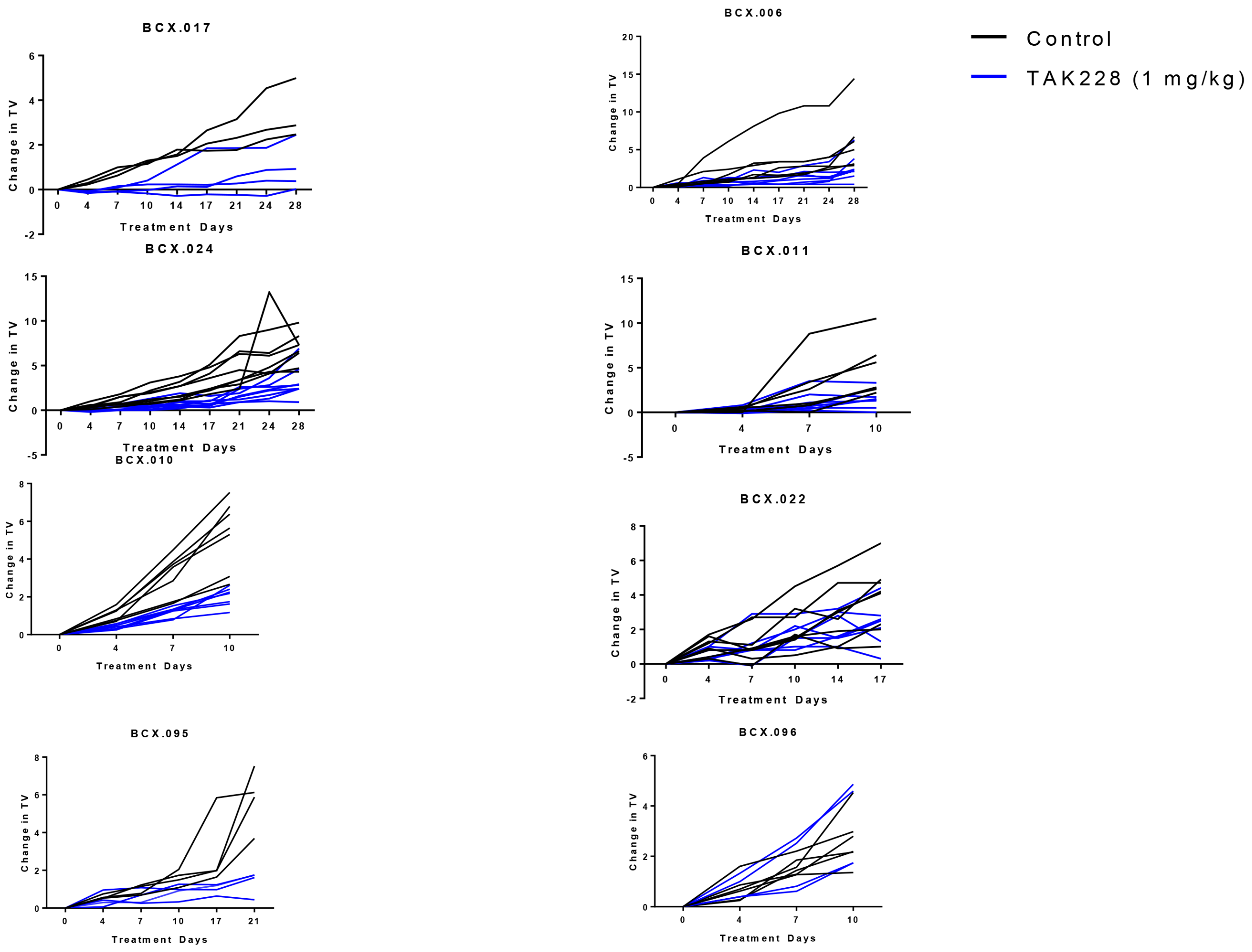

Supplementary Figure 1: Breast cancer xenografts display differential sensitivity to TAK228.

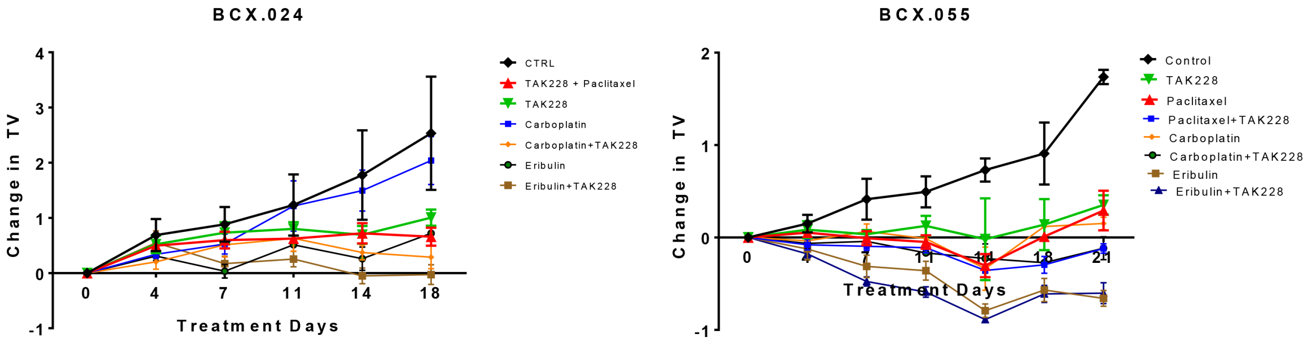

Supplementary Figure 2: Effects of TAK228 in combination with chemotherapy *in vivo*.

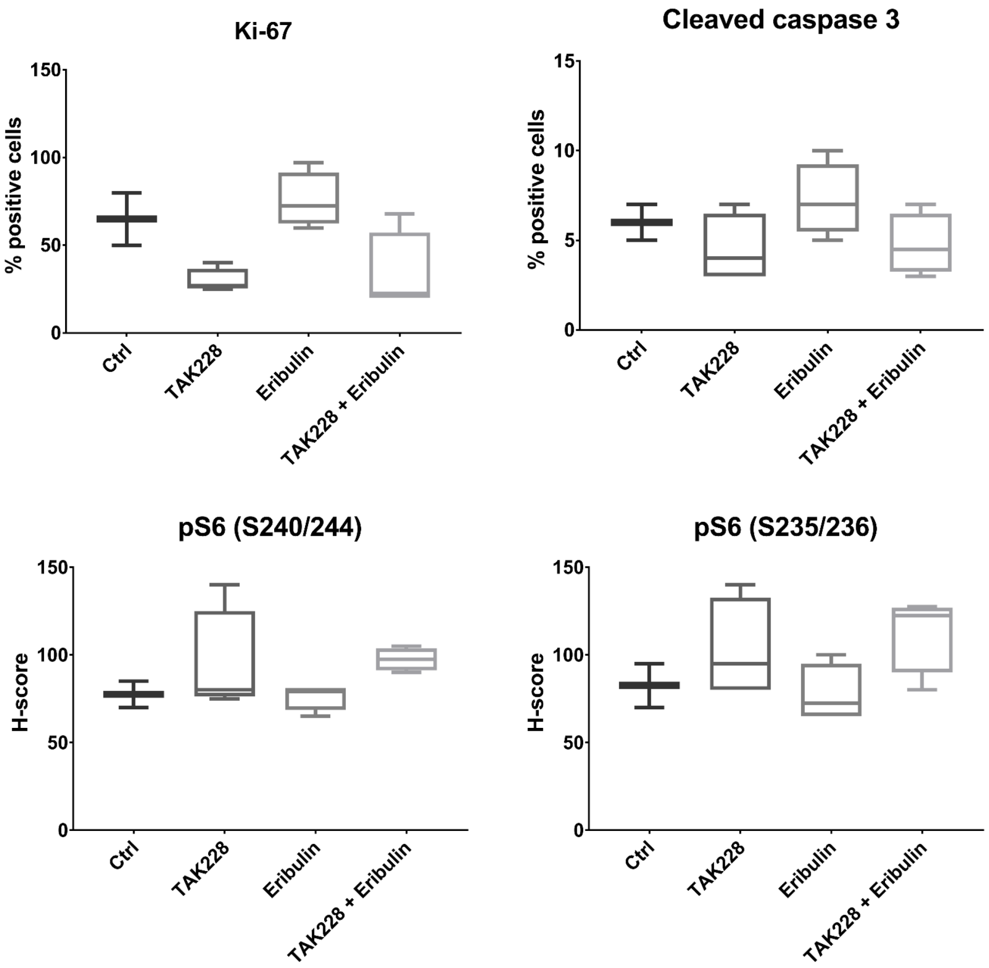

Supplementary Figure 3: Immunohistochemical analysis of BCX.024 patient-derived xenograft model. Control (*n* = 2), TAK228 (*n* = 4), eribulin (*n* = 4), and TAK228-eribulin combination (*n* = 4) tumors were assessed by IHC for Ki-67, cleaved caspase 3, pS6 (S235/236), and pS6 (S240/244) expression. The box extends from the 25th to 75th percentiles and the line in the middle plotted at the median. Whiskers are drawn min to max, showing all points.
